# The relationship between foot posture, body mass, age and ankle, lower-limb and whole-body flexibility in healthy children aged 7 to 15 years

**DOI:** 10.1186/s13047-016-0144-7

**Published:** 2016-04-27

**Authors:** Fiona Hawke, Keith Rome, Angela Margaret Evans

**Affiliations:** School of Health Sciences, University of Newcastle, Ourimbah, NSW Australia; Health & Research Rehabilitation Institute, School of Podiatry, AUT University, Auckland, New Zealand; Discipline of Podiatry, College of Science, Health, and Engineering, La Trobe University, Victoria, 3086 Australia

**Keywords:** Foot posture, Children, Body mass, Flexibility

## Abstract

**Background:**

The complex relationship between foot posture, flexibility, body mass and age in children is not well understood. The objectives of this *post hoc* analysis were to explore the relationships between foot posture, flexibility, body mass in children aged seven to 15 years.

**Methods:**

Thirty healthy, asymptomatic children (20 girls, 10 boys) aged 7 to 15 years with a mean age (SD) of 10.7 (2.3) years, were recruited through the Auckland University of Technology (AUT) Podiatry Clinic, Auckland, New Zealand. Clinical data were collected by a podiatrist with 20 years’ experience and included: height and weight (for Body Mass Index), Foot Posture Index-6 (FPI), Beighton score, Lower Limb Assessment Scale score (LLAS); and ankle lunge angle. For this *post hoc* analysis, Pearson’s test and Spearman’s rho were used to explore relationships between variables. Statistical significance level was *p* < 0.05.

**Results:**

Data for each of the 30 participants for each variable were included in analyses, which returned the following statistically significant results: higher FPI was associated moderately with higher Beighton score (*r* = 0.44, *p* = 0.01); greater lunge angle was associated moderately with higher Beighton (*r* = 0.40, *p* = 0.02) and LLAS (*r* = 0.42, *p* = 0.02) scores; older age was associated strongly with higher BMI (*r* = 0.52, *p* = <0.01) and moderately with lower Beighton (*r* = −0.41, *p* = 0.024) and LLAS (*r* = −0.40, *p* = 0.03) scores; and higher Beighton score was associated strongly with higher LLAS (*r* = 0.85, *p* = <0.01). There was no difference in foot posture between girls and boys (*p* = 0.21).

**Conclusions:**

In this sample of healthy, asymptomatic children age 7 to 15 years, children with a more pronated foot type exhibited greater lower limb and whole-body flexibility, but not greater ankle joint flexibility. There was strong agreement between lower-limb and whole-body flexibility. This study highlights the importance of assessing the paediatric flat foot in the context of a developing body.

## Background

The complex relationship between foot posture, flexibility, body mass and age in children is not well understood. As discrete factors, age, body mass, and joint flexibility have each been associated with variance in paediatric foot posture [1–3]. In a study of 835 Austrian children age three to six years, Pfeiffer [1] reported that age, body weight, and gender correlated with flat foot posture; with younger, heavier boys having flatter feet. Similarly, a study of 1598 children of the same age in Taiwan found correlation between age, body weight, gender, joint flexibility, habitual W-sitting and flat foot posture [2]. This study illustrated that bilateral flat foot posture reduces with the child’s age, but may be retained or increased in boys who are overweight for their age, have increased joint flexibility, and/or sit in the W-position [2].

Some critique of these studies is in order given the young ages of the children examined (when flatfoot is morphologically expected) and given the rudimentary methods of identifying flatfeet, which were not reportedly tested for repeatability. Both the Pfeiffer [1] and Chen [2] studies identified flatfoot by the visualised appearance of the medial arch, supplemented by heel position in the Pfeiffer study [1]. Of the young children examined, Pfeiffer [1] identified flatfoot in 44 %, and Chen identified bilateral flatfoot decreasing with age from 55 % at age 3 years to 21 % at age 6 years. Chen [2] visually identified children as having flat or normal feet, and then subdivided children with flat foot posture into bilateral and unilateral presentations, and, in the latter, age and obesity were not associated with the child’s unilateral flat foot posture. The difference in bilateral and unilateral flatfoot presentations deserves attention, as the findings were discrepant. While Chen [2] found reducing prevalence of bilateral flatfoot from 3 to 6 years of age, unilateral flatfoot was detected in 14 % of three year old children, and 18 % of children aged six years [2]. Given the criteria by which foot posture was assessed and the age of the children when they are seldom still, these findings may be imprecise. Interestingly, the same authors did not address laterality in two subsequent studies of the same cohort, having then adopted less subjective footprint analysis of foot posture instead of visual assessment [3, 4].

A larger Taiwanese investigation of 2083 older children aged 7 to 12 years, also found correlation between flatfoot and age, gender and body weight [5]. This study used static footprints to assess foot posture, and then categorised the visual appearance of the footprints: normal and grades 1 to 3 indicating increasing medial arch loading, surmised to indicate a flatter foot. Acquiescent with previous studies [1, 2] flatter feet were most prevalent in younger, overweight/obese boys, but clearly the method of foot posture assessment differed as did the age group of the children examined. Chang [5] also found reducing flatfoot with age (69.8 % at age 7 years to 39.0 % at age 12 years), but in a non-linear manner. Given the subjective classification system of the footprints, and the combining of moderate and severe categories, the prevalence of flatfoot as presented by Chang et al. [5] is perhaps less useful than assessing the associations between the individual gradings of foot posture and factors such as age, BMI and gender. Many of the different grading and scoring systems for flatfeet are largely subjective. In contrast, the FPI is a superior method with gradings identified in a more rigorous way.

The relationship between paediatric foot posture and the correlates of body mass, joint flexibility, age and gender have been investigated [2, 5–8], but using variably defined methods in children whose ages have differed. The objectives of this *post hoc* analysis were to: (1) explore, using validated and reliable methods, the relationships between foot posture, flexibility, body mass in children aged seven to 15 years in Auckland, New Zealand; and (2) explore difference in foot posture between girls and boys in the same sample.

## Methods

Between February 2011 and March 2012, a convenience sample of thirty healthy, asymptomatic children with no history of foot injury or surgery, and not reporting current foot pain, aged between 7 and 15 years were recruited through the Auckland University of Technology (AUT) Podiatry Clinic, Auckland, New Zealand. The AUT Ethics Committee approved the study (approval number 10/291) and parents/guardians provided written informed consent.

Age, gender and ethnicity were recorded to characterise the sample. Clinical data collected were: Body Mass Index, left Foot Posture Index-6 (FPI) [9], Beighton Scale score [10], Lower Limb Assessment Scale score (LLAS) [11]; and left ankle lunge angle [12]. The order of physical measures was consistent among participants. One podiatrist with 20 years’ experience (AE) performed all tests. We demonstrated excellent test-retest reliability (intra-class correlation coefficient >0.85 [mean 95 % CI 0.86–0.97]) for all clinical measures [7, 13].

### Procedure

FPI-6 for the left foot only [14] was measured following a published protocol [9]. FPI was measured after the patient took five or more steps on the spot and came to rest in a comfortable standing position with arms by their sides and looking straight ahead. Each foot was scored using six criteria: (1) talar head palpation; (2) curves above and below the lateral malleolus; (3) inversion/eversion of the calcaneus; (4) bulge in the region of the talonavicular joint; (5) congruence of the medial longitudinal arch; and (6) abduction/adduction of the forefoot on the rearfoot. Each criterion was given a score between–2 and 2, where scores less than zero indicate a supinated alignment and scores greater than zero indicate a pronated alignment. Scores of all criteria were added together to create an overall score for each foot from–12 (most supinated) to +12 (most pronated).

The Beighton scale [10] was rated to ascertain the presence of joint hypermobility at the wrist, fifth metacarpal phalangeal joint, elbow, knee (all bilateral and non-weight-bearing) and the lumbo-sacral spine (forward flexion, in stance). The Beighton scale yields a score out of 9-points, whereby the arbitrary cut-off of 5/9 or greater conventionally indicates joint hypermobility [10].

The LLAS [11] was assessed to gauge joint hypermobility of the lower limb. One point is awarded per limb for each of the following: (1) hip flexion where the anterior thigh contacts the chest; (2) hip abduction where the lateral femoral condyles touch the plinth; (3) knee hyperextension where the heels lifts >3 cm from the plinth when the foot is lifted when in a long sitting position; (4) positive knee anterior draw test; (5) > 1 cm medial or lateral, or > 2 cm overall rotation of the tibia at the knee; (6) >15° ankle dorsiflexion when the knee is flexed; (7) positive ankle anterior draw test; (8) >45° subtalar joint inversion with lateral prominence of the talar head assessed non-weight-bearing; (9) >45° midtarsal joint inversion; (10) >1 cm midtarsal abduction/dorsiflexion and adduction/plantarflexion; (11) >90° 1st metatarsophalangeal joint dorsiflexion; (12) subtalar joint at end range of pronation when weightbearing. Each limb yields a final score out of 12-points, whereby the cut-off of 7/12 or greater conventionally indicates joint hypermobility [11]. In this study only the left leg was assessed, and given a score out of 12-points.

Weight-bearing ankle dorsiflexion range of the left limb only [14] was assessed using the Lunge test [12, 15], a weight-bearing measure of ankle (talocrural joint) dorsiflexion range when the knee is flexed. The participant stood on a solid, horizontal surface facing a solid, vertical wall with both hands resting on the wall for support. The testing foot was placed perpendicular to the wall (to limit dorsiflexion through subtalar and midfoot joints), and the contralateral foot was placed in a comfortable, stable position. The test involved the participant lunging the knee as far forward as possible over the foot whilst maintaining the heel on the floor. At the maximum lunge point, the investigator recorded the angle of the tibia to the vertical as a measure of ankle dorsiflexion using a digital inclinometer (Smart Tool™) applied to the anterior surface of the tibia.

### Data analysis

Data were transcribed to SPSS version 20 (SPSS, Inc., Chicago, IL, USA). As this is a post hoc analysis of existing data, no power calculation was conducted. Descriptive statistics were used to characterise the sample.

Age, BMI, ankle lunge, Beighton score and LLAS were analysed as continuous data. Normality and symmetry was assessed using histograms and the Kolmogorov-Smirnov test. Relationships between continuous variables were explored with Pearson’s point-biserial correlation coefficient for normally distributed continuous data and Spearman’s rho for non-normally distributed continuous data. As FPI raw scores were normally distributed (Kolmogorov-Smirnov test *p* = 0.08; mean and median within 10 %; skewness and kurtosis values between–1 and 1) FPI was also analysed as continuous data rather than as categorical data [9]. An independent sample *t*-test with statistical significance level of *p* < 0.05 was conducted to explore difference in foot posture between girls and boys. The magnitude of effect sizes was estimated using the following parameters suggested by Cohen [16]: small (weak) = 0.1, medium (moderate) = 0.3 and large (strong) = 0.5. Due to limitations imposed by the sample size, regression analysis to determine dependence and independence of relationships was not performed [17].

## Results

Thirty children (20 girls, 10 boys) age 7 to 15 years with a mean (SD) of 10.7 (2.3) years participated. Ethnicity was reported by parent/guardian as Caucasian for 27 (90 %) children, Asian for 2 (7 %) children and Maori for 1 (3 %) child. Descriptive statistics are presented in Table 1 and the strength and direction of relationships between variables are presented in Table 2.

**Table 1:** Demographic Characteristics

Category	Score
BMI (Kg/m^2^), mean (SD)	18.2 (3.4)
FPI, mean (SD)	2.8 (2.3)
Lunge (^0^), mean (SD)	41.1 (7.1)
Age (years), mean (SD)	10.7 (2.3)
Beighton, median (IQR)	2.5 (5)
LLAS, median (IQR)	6.0 (12)

**Table 2:** Relationship Between Variables (R-value and *p*-value)

	BMI	FPI	Lunge	Beighton	LLAS
FPI	0.14 (0.48)		.		
Lunge	−0.03 (0.88)	0.17 (.36)			
Beighton	−0.22 (0.24)	0.44 (0.01) ^a^	0.34 (.03) ^a^		
LLAS	−0.34 (0.06)	0.27 (0.16)	0.42 (0.02) ^a^	0.85 (<.000) ^a^	
Age	0.52 (.003) ^a^	−0.01 (0.98)	−0.05 (0.79)	−0.41 (0.02) ^a^	−0.40 (0.03) ^a^

^a^ Statistically significant correlation at 5 % level

Higher FPI was associated with higher Beighton score (*r* = 0.44, *p* = 0.01). Greater lunge angle was associated with higher Beighton (*r* = 0.40, *p* = 0.02) and LLAS (*r* = 0.42, *p* = 0.02) scores. Older age was associated with higher BMI (*r* = 0.52, *p* = <0.01) and lower Beighton (*r* = −0.41, *p* = 0.02), and LLAS (*r* = −0.40, *p* = 0.03) scores. Higher Beighton score was associated with higher LLAS (*r* = 0.85, *p* = <0.01). There was no statistically significant association between FPI with BMI (*r* = 0.14, *p* = 0.48), ankle lunge (*r* = 0.17, *p* = 0.36), LLAS (*r* = 0.27, *p* = 0.16) or age (*r* = −0.01, *p* = 0.98). The difference between Foot Posture Index total scores for girls with a mean (SD) of 2.5 (2.5) and boys with a mean (SD) of 3.6 (1.8) was not significant (p = 0.21).

## Discussion

The findings were commensurate with previously reported investigations [1, 2], in that a relationship was found between flatter foot posture and joint flexibility. In contrast, there was no relationship detected between foot posture and age, body mass index or gender.

This study agreed with several previous investigations in finding that children with a flatter foot posture exhibited greater lower limb and whole-body flexibility [4, 5]. The findings of this study also indicate that increased flexibility is correlated between three reliable clinical measures: LLAS, Beighton scale, and the ankle lunge [6]. However, whilst flatfoot correlated with whole body and lower limb flexibility as detected by the Beighton scale and the LLAS respectively, there was no significant relationship between flatfoot and increased flexibility of ankle lunge. In essence, it appears that whole body hypermobility may be associated with increased ankle dorsiflexion and flatfoot. The relationship between foot pronation and joint flexibility may need to be considered in children who are presenting with symptomatic flatfoot.

This study found strong agreement between lower-limb and whole-body flexibility, and medium strength agreement between ankle flexibility with lower-limb and whole-body flexibility. The lesser strength of association between ankle flexibility and the other measures of flexibility may be due to the functional demands placed on the calf musculature in gait, which differs between children depending on their gait style (for example, increased demand and possible subsequent calf tightness in toe walking). Importantly, there was no notable association between foot posture and ankle flexibility.

Within this convenience sample of normal children, the older children exhibited less lower limb and whole-body flexibility, but not less ankle joint flexibility. Given the small sample size and defined age range within childhood, this may indicate that ankle joint range remains static at these ages, or that this finding was specific to this cross-sectional set of observations, rather than generalizable. Given the morbidity associated with hypermobility which may persist across the lifespan [18], it is important that clinicians recognise and appreciate the impact of this presentation, which is too often disregarded as being benign or even advantageous [19]. Whilst there was no significant difference detected between foot posture and gender in this study, the small sample size may have underpowered analysis. However, there is the supported expectation that the foot posture of children across the bounds of the age group of this study (7 to 15 years) will vary; less so in those aged 10–15 years, and more so in those aged 7–10 years [5, 20]. Clinically children in their first decade are more likely to have flatter feet when younger, overweight and male [21, 22]. The adult foot posture and less variation is expected after age 10 years, where flatfeet are less common, or may be associated with specific physiology viz. connective tissue hypermobility [23].

This is a cross-sectional study so no inferences should be made about cause-effect relationships. The convenience sample included predominately Caucasian children who were all asymptomatic and between the ages of 7 and 15 years. Gender distribution was uneven. Results should not be generalised outside of this population, for example to adults, younger children or symptomatic people. FPI, Beighton score and LLAS data were statistically analysed using non-parametric tests. Children with unilateral flatfoot were not excluded. This should be considered when planning eligibility criteria for future studies.

There is a growing evidence base for factors associated with flat foot in children. There is a need for prospective observational studies to investigate potential predictors of flatfeet in children. Additional studies spanning from childhood, through adolescence to adulthood will provide important information about the evolution of flatfoot and strengthen the evidence base for decisions about treatment of children with asymptomatic flat feet.

## Conclusion

In this sample of healthy, asymptomatic children age 7 to 15 years, children with a more pronated foot type exhibited greater lower limb and whole-body flexibility, but not greater ankle joint flexibility. Older children exhibited less lower-limb and whole-body flexibility, but not ankle joint flexibility. Higher body mass index was not associated with a more pronated foot type and there was no important relationship between age and foot posture. There was strong agreement between lower-limb and whole-body flexibility. A large longitudinal study is required to explore potential predictors of foot posture throughout development and to investigate the independence of relationships between variables. Such an investigation needs to incorporate measures of foot posture which are demonstrably robust.

## Abbreviations

BMI: body mass index
FPI: foot posture index-6
LLAS: lower limb assessment scale score
SD: standard deviation
